# Hypercalcemia and Bone Metastasis in a Case of Large Cell Neuroendocrine Carcinoma With Unknown Primary

**DOI:** 10.1155/2024/8792291

**Published:** 2024-05-21

**Authors:** Ekrem Yetiskul, Jordyn Salak, Fatema Arafa, Alaukika Agarwal, Amanda Matra, Muhammad Niazi, Marcel Odaimi

**Affiliations:** ^1^Department of Internal Medicine, Staten Island University Hospital, 475 Seaview Avenue, Staten Island, New York 10305, USA; ^2^Department of Hematology & Oncology, Staten Island University Hospital, 475 Seaview Avenue, Staten Island, New York 10305, USA

## Abstract

Large cell neuroendocrine carcinoma (LCNEC) constitutes a rare subset of highly undifferentiated malignancies known for their aggressive nature. Although these tumors commonly originate in the lungs and gastrointestinal tract, their potential occurrence is not restricted to specific anatomical sites, giving rise to a variety of symptoms. Notably, cases of neuroendocrine tumors (NETs) with an unidentified primary source exhibit a graver prognosis and shorter survival periods compared to those with clearly identified origins. NETs frequently demonstrate a propensity to metastasize, spreading to diverse anatomical regions such as the liver, lungs, lymph nodes, and bones, illustrating their aggressive nature and the complexity of their management. In this context, we present the case of a 59-year-old male who sought medical attention in the emergency department due to right upper quadrant (RUQ) abdominal pain. Initial diagnostic assessments revealed significantly elevated liver function tests and severe hypercalcemia. A right upper quadrant ultrasound (RUQ US) was subsequently performed, which revealed heterogeneous hepatic echotexture with innumerable echogenic masses, suggesting a metastatic process. A computed tomography (CT) scan was then ordered to evaluate further the RUQ US findings, which showed numerous hypovascular liver masses, raising concerns of malignancy. A liver biopsy confirmed a diagnosis of LCNEC with an unidentified primary source.

## 1. Introduction

Neuroendocrine tumors (NETs) are rare tumors with cells that share markers of both endocrine and neuronal differentiation, including secretory granules and hormone production. The current incidence rate for NETs in the United States stands at 6.25 per 100,000 individuals, which continues to rise annually [1, 2]. The primary site of origin cannot be identified by routine imaging or histopathology in 12%–22% of cases [3]. Primary unknown NETs have a worse prognosis and shorter survival than other NETs, with limited data available in the literature concerning this subgroup [4]. Large cell neuroendocrine carcinoma (LCNEC) is a rare subgroup of high-grade neuroendocrine cancer that can occur throughout the body. A retrospective analysis performed by Jules et al. discovered that out of 383 patients with stage IV LCNEC, metastasis most commonly occurred in the liver (47%), bone (32%), brain (23%), adrenal gland (19%), lung (14%), pleura (7%), and extrathoracic lymph nodes (16%) [5]. Although bone metastasis can occur in NETs, hypercalcemia caused by bone metastasis in patients with NETs was noted to be 3% [6]. We present a case of unknown LCNEC with diffuse bone metastasis, which resulted in severe hypercalcemia. Furthermore, the report delves into the complexities of LCNEC, emphasizing its rarity and diverse presentation across various anatomical sites.

## 2. Case Presentation

We present a case of a 59-year-old male with a past medical history of traumatic brain injury (TBI), hypertension (HTN), hyperlipidemia (HLD), and seizure disorder who presented to the emergency department due to right upper quadrant (RUQ) abdominal pain, which started 2 days prior to presentation. The patient further described his RUQ abdominal pain as dull, constant without radiation, and without associated symptoms, including nausea and vomiting. He also denied any alleviating factors and specific triggers that exacerbate pain, including the consumption of fatty foods. Upon physical examination, there was prominent and tender hepatosplenomegaly, but there was no evidence of jaundice. The patient's social history is significant for a 20-year pack history of smoking; he denies alcohol consumption, denies any history of malignancy in his family, and has worked as a delivery driver for the past 20 years.

**Figure 1:**
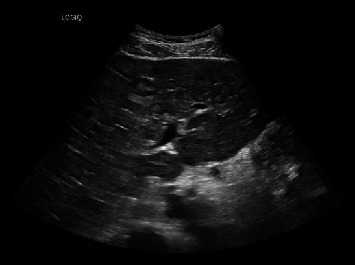
Right upper quadrant ultrasound demonstrated heterogeneous hepatic echotexture with innumerable echogenic masses, suggesting a metastatic process.

**Figure 2:**
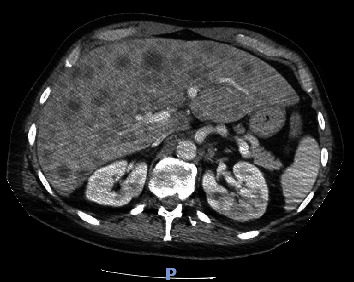
Axial section obtained from computed tomography of the abdomen and pelvis shows numerous hypovascular liver masses measuring up to 4.8 cm.

**Figure 3:**
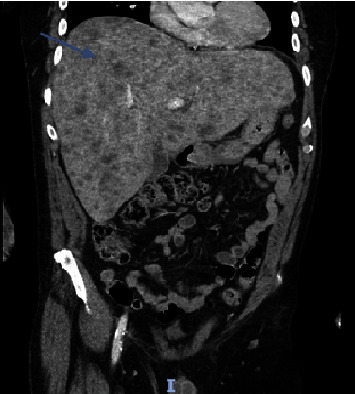
Coronal section obtained from computed tomography of the abdomen and pelvis shows numerous hypovascular liver masses measuring up to 4.8 cm (blue arrow).

**Figure 4:**
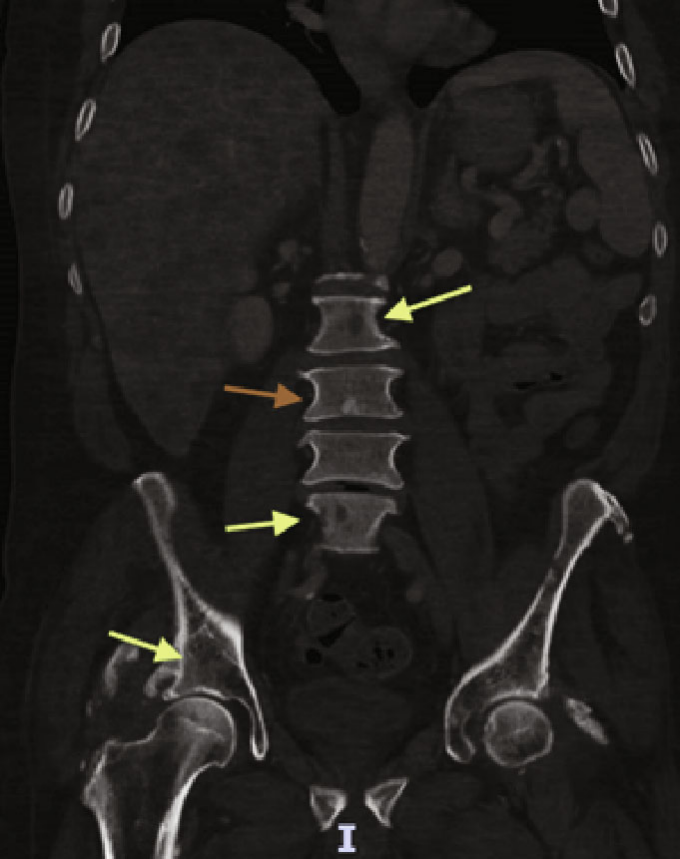
Coronal sections obtained from computed tomography of the abdomen and pelvis show sclerotic (orange arrow) and lucent foci (yellow arrows) within the iliac bones and vertebral bodies.

Laboratory findings (Table 1) were significant for leukocytosis of 11.98 K/UL, potassium level of 5.6 mmol/L, calcium level of 14.9 mg/dL (corrected for albumin), albumin level of 4.4 g/dL, alkaline phosphatase level of 403 U/L, aspartate aminotransferase (AST) level of 285 U/l, alanine aminotransferase (ALT) level of 224 U/l, magnesium level of 1.4 mg/dL, uric acid level of 13.4 mg/dL, and serum phosphorus level of 1.9 mg/dL. Right upper quadrant ultrasound (RUQ US) demonstrated heterogeneous hepatic echotexture with innumerable echogenic masses, suggesting a metastatic process (Figure 1). Mild gallbladder wall thickening was noted, deemed nonspecific in the context of liver disease, with limited gallbladder evaluation due to the described findings. Cholelithiasis versus gallbladder sludge was also noted. Contrast-enhanced abdominal/pelvic computed tomography (CT A/P) revealed numerous hypovascular liver masses measuring up to 4.8 cm, along with sclerotic and lucent foci within the iliac bones, vertebral bodies, and partially imaged sternum, raising concerns for metastatic disease (Figures 2–4). The patient received zoledronic acid, calcitonin, and intravenous fluids and was subsequently admitted to the hospital for evaluation for metastatic disease with an unknown primary, acute hepatitis, and severe hypercalcemia. A diagnostic workup of hypercalcemia was performed which showed an intact parathyroid hormone (PTH) level of 11 pg/mL (reference range 15–65 pg/mL), parathyroid hormone-related peptide (PTHrP) level of < 2.0 pmol/L (reference range < 2.0 pmol/L), 25-hydroxy vitamin D level of 19 pg/mL (reference range 19.9–79.3 pg/mL), and 1,25-hydroxy vitamin D level of 20 ng/mL (reference range 30–80 ng/mL) (Table 2). Given the results of the hypercalcemia laboratory evaluation, it was determined that the patient's hypercalcemia was secondary to bone metastasis.

**Figure 5:**
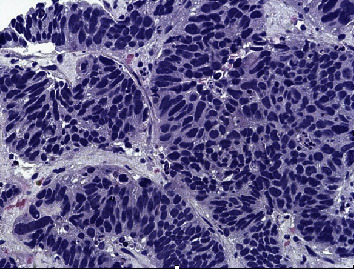
Morphologic features and immunoprofile are consistent with LCNEC. The origin of tumor could not be determined by IHC analysis. Neuroendocrine carcinomas may be TTF-1 positive, regardless of origin. Few cells weakly positive for CDX-2 suggest an appendix, pancreas, or intestinal primary. Immunohistochemistry showed positive CK7, AE1/AE3, synaptophysin, chromogranin, CD56, TTF-1 and p63 (scattered cells, weak), CDX-2 (few cells, weak), and Ki-67 proliferation index (99%).

**Figure 6:**
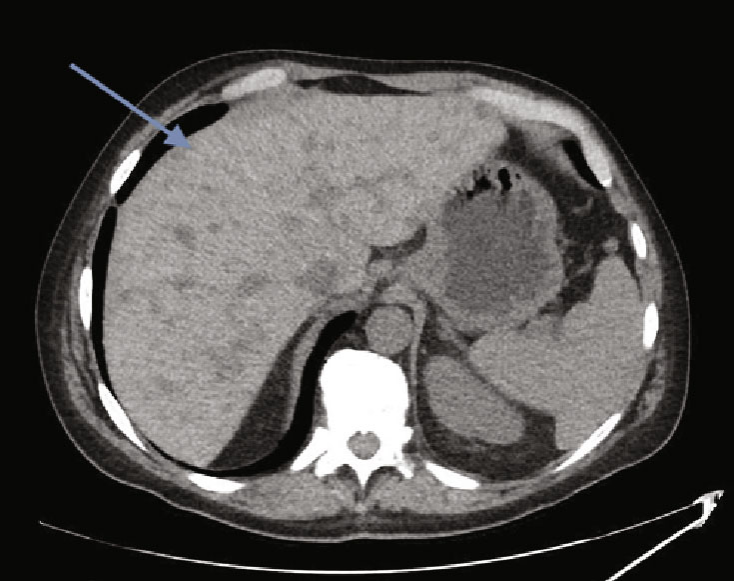
CT abdomen and pelvis was performed 2 months after starting chemotherapy which demonstrated a slight decrease in the size of hepatic metastatic lesions, which may represent a partial response to therapy (blue arrow).

Further diagnostic evaluation included further radiologic examinations to assess for metastatic disease. Computed tomography of the head (CT head) did not reveal any evidence of metastatic disease, but magnetic resonance imaging of the brain (MR brain) was suggested for further evaluation. MR brain did not reveal any metastatic disease. Computed tomography of the chest (CT chest) showed an indeterminate grouping of nodules/lobular nodules in the superior right lower lobe spanning 1.5–2 cm. Neoplastic or metastatic processes could not be excluded. Interventional radiology was consulted for biopsy, and specimens were obtained from the patient's liver. The pathology report showed LCNEC; however, the origin of the tumor could not be determined by immunohistochemical (IHC) analysis. Immunohistochemistry results were positive for CK7, AE1/AE3, synaptophysin, chromogranin, CD56, TTF-1 and p63 (scattered cells, weak), and CDX-2 (few cells, weak), suggesting possible appendiceal, pancreatic, or intestinal primary; Ki-67 proliferation index was 99%, suggesting aggressive behavior (Figure 5). IHC analysis was negative for CK20, p40, CK5/6, PAX8, NKX3.1, hepatocyte marker, and napsin A. Programmed death-ligand 1 (PD-L1) IHC analysis showed tumor proportion score (TPS) of 0%. Next-generation sequencing (NGS) testing was sent on the tumor sample, but it was unable to reveal any genetic mutation of clinical significance except a few variants of unknown significance (VUS). The tumor's microsatellite status (MSS) was stable. Tumor markers obtained during the hospital course were significant for a carcinoembryonic antigen (CEA) level of 5002 ng/mL (reference range 0.0–3.8 ng/mL), cancer antigen 19-9 (CA 19-9) level of 1393 U/mL (reference range < = 35 U/mL), and serum chromogranin level of 1653 ng/l (reference range < 39 ng/l). Chemotherapy was initiated, which consisted of carboplatin and etoposide, given the neuroendocrine origin of the tumor. Of note, positron emission tomography (PET) was not performed prior to the initiation of chemotherapy. So far, the patient has received three cycles of chemotherapy, and he has tolerated it without any complications. The patient has improved functionally, and his hypercalcemia has resolved after one dose of each zoledronic acid and calcitonin. A repeat CT of the patient's chest, abdomen, and pelvis was performed 2 months after starting chemotherapy, which demonstrated a slight decrease in the size of hepatic metastatic lesions, which may represent a partial response to therapy (Figure 6).

## 3. Discussion

NETs are a relatively rare type of heterogeneous tumors that occur in the secretory cells of the diffuse neuroendocrine system [7]. They are characterized by a relatively slow growth rate and the ability to secrete various peptide hormones and biogenic amines [7]. Although NETs were historically regarded as rare cancers, there has been a significant surge in their detected incidence over recent decades [6]. NETs of unknown origin account for more than 10% of all NETs, and most of these tumors are poorly differentiated and, therefore, very aggressive [8]. Neuroendocrine cancers constitute less than 5% of all cancers of unknown primaries [9]. These tumors have a wide range of histologic appearance, biologic behavior, and response to treatment. World Health Organization (WHO) has classified well-differentiated gastroenteropancreatic NETs into low-grade (G1), intermediate-grade (G2), and high-grade (G3) categories based on proliferative rate (as assessed by proliferative index by Ki-67 index) and histologic appearance. G1 and G2 generally have an indolent disease course. All poorly differentiated G3 NETs have an aggressive clinical course (Klimstra DS, Kloppell G, La Rosa S, Rindi G. Classification of neuroendocrine neoplasms of the digestive system) [10]. It is essential to differentiate between low-grade G1, well-differentiated indolent neoplasms, and high-grade G3, poorly differentiated, highly aggressive neoplasms, since this changes the management approach. NETs are often diagnosed at Stage IV, with the liver and peritoneum being the most common sites of metastasis [11]. Skeletal colonization is often regarded as a rare event in patients with NETs, and retrospective series reported the incidence of bone metastases as high as 20% in patients with advanced disease [11]. The most common sites of bone metastasis in NET patients are the axial skeleton, mostly at vertebra levels, followed by the pelvic region and ribs [6]. Locating the primary tumor in a NET of unknown primary can be challenging because of its occult nature and the small size of the primary lesion. These tumors tend to have potentially diverse anatomical origins (gastrointestinal, lung, and pancreas), and their aggressive nature with a high proliferative index makes it even more intricate to detect them in the early stage and treat them with curative intent. A 92-gene MCCA (molecular classification of cancer assay) can sometimes assist in the identification of the primary source of the tumor [12]. The use of advanced diagnostic imaging techniques like PET, CT scan, MRI, endoscopic procedures, and somatostatin receptor scintigraphy can assist in the detection of primary tumors in such scenarios [13]. PET scan is preferred over somatostatin receptor imaging in metastatic settings because of the high metabolic activity of these tumors and the relatively decreased sensitivity of the latter modality [14]. MRI of the brain is recommended in metastatic settings because of the increased propensity of these tumors to intracranial spread [14].

Hypercalcemia is defined as an increase in the serum calcium level above the upper limit of normal for a given reference value used in a laboratory [15]. The differential diagnosis of hypercalcemia includes multiple pathologic entities, but the most common etiologies include hyperparathyroidism and hypercalcemia of malignancy [15]. Hypercalcemia of malignancy is a common finding in patients with cancer, affecting up to 44.1% of patients [15]. There are several mechanisms of hypercalcemia of malignancy, including the production of PTHrP, osteolytic metastasis, ectopic activity of 1-alpha-hydroxylase, the formation of 1,25-dihydroxycholecalciferol, and the ectopic production of PTH [16]. Approximately 80% of malignancy-related hypercalcemia is mediated by the production of PTHrP, and approximately 20% of malignancy-related hypercalcemia is caused by osteolytic metastases [16]. Although bone metastasis can occur in as many as 20% of patients with advanced NETs, hypercalcemia was rarely described in NET patients with bone metastasis and has been reported in up to 3% of patients [6, 11]. Our patient with diffuse bone metastases to his iliac bones, vertebral bodies, and sternum was evaluated for the cause of hypercalcemia. Given the findings of low intact PTH, low 25-hydroxy vitamin D, low 1,25-hydroxy vitamin D, and normal PTHrP levels, it was determined that osteolytic metastases caused his hypercalcemia.

The presence of bone metastases affects the clinical course and prognosis of NET patients, but its role in defining therapeutic strategies has yet to be clarified [6]. High response rates close to 66% have been observed with platinum-based chemotherapy doublets (carboplatin or cisplatin with etoposide) in poorly differentiated NETs with well-defined primary sites. This data has been extrapolated to NETs of unknown primary and has shown similar results [17, 18]. A response rate as high as 70% has been observed in one of the most extensive published studies of 99 patients treated with an etoposide/platinum-based chemotherapy regimen [9]. The role of immune checkpoint inhibitors (ICI) remains under investigation in this setting.

Nevertheless, ICI has shown its efficacy in neuroendocrine skin tumors (Merkel cell carcinoma) [19, 20]. Snoeck et al. studied the efficacy of temozolomide, an alkylating agent, with bevacizumab, a monoclonal antibody against vascular endothelial growth factor (VEGF), in patients who progress on platinum-based chemotherapy [21]. Case reports of good response are observed with mammalian targets of rapamycin (mTOR) inhibitors like everolimus in patients with stable disease for 6 to 15 months [22]. Bone metastases often occur alongside metastases in other distant locations, which complicates the decision-making process regarding whether the involvement of bones in metastasis warrants a particular treatment approach or necessitates changes in the treatment plan for NETs [6]. Currently, there is no consensus regarding the management of bone metastases from NETs, and more research is needed to ascertain if early identification of bone metastases can prevent complications like hypercalcemia, immobility, and disability, thereby influencing changes in therapeutic approaches [23]. Given the uncommon and diverse nature of NETs and the fact that bone metastases only occur in a fraction of these patients, there may never be a specialized trial to assess the effectiveness of these drugs in delaying or preventing bone metastases [23]. Consequently, clinicians often rely on information derived from studies on other solid tumors or retrospective studies on NETs to guide their practice [23]. In the case of our patient, his treatment regimen included carboplatin and etoposide, which was extrapolated from small cell lung cancer experience as there are no guidelines for the presented case.

## 4. Conclusion

NETs, though historically rare, have seen a rising incidence in recent decades. Hypercalcemia, a common complication in various cancers, is less frequently observed in NET patients with bone metastases. The case highlights the complexity of diagnosing and managing hypercalcemia in patients with NETs, mainly caused by osteolytic metastases. The occurrence of bone metastases in NET patients impacts their clinical course and prognosis and presents challenges in defining effective therapeutic strategies. The lack of consensus and randomized trials on managing NETs of unknown primary underscores the need for more research for more tailored treatment approaches. The evolving understanding of NETs, their metastatic patterns, and associated complications like hypercalcemia necessitate a multidisciplinary approach for optimal patient management. It highlights the importance of continued research in this field.

## Figures and Tables

**Table 1:** Laboratory findings on presentation.

White blood cell count (WBC)	11.98 K/*μ*L (reference range: 4.8–10.8 K/*μ*L)
Potassium, serum	5.6 mmol/L (reference range: 3.5–5.0 mmol/L)
Calcium, serum	15.2 mg/L (reference range: 8.4–10.8 mg/dL)
Magnesium, serum	1.4 mg/L (reference range: 1.8–2.4 mg/dL)
Phosphorus, serum	1.9 mg/L (reference range: 2.1–4.9 mg/dL)
Albumin, serum	4.4 g/dL (reference range: 3.5–5.2 g/dL)
Alkaline phosphatase	403 U/L (reference range: 30–115 U/L)
Aspartate aminotransferase (AST)	285 U/L (reference range: 0–41 U/L)
Alanine aminotransferase (ALT)	224 U/L (reference range: 0–41 U/L)
Uric acid, serum	13.4 mg/L (reference range: 3.4–8.8 mg/dL)

**Table 2:** Hypercalcemia laboratory findings.

Intact parathyroid hormone (intact PTH)	11 pg/mL (reference range 15–65 pg/mL)
Parathyroid hormone-related peptide (PTHrP)	< 2.0 pmol/L (reference range < 2.0 pmol/L)
25-Hydroxy vitamin D	19 pg/mL (reference range 19.9–79.3 pg/mL)
1,25-Hydroxy vitamin D	20 ng/mL (reference range 30–80 ng/mL)
